# Correction: Evolution of reduced co-activator dependence led to target expansion of a starvation response pathway

**DOI:** 10.7554/eLife.84187

**Published:** 2022-10-20

**Authors:** Bin Z He, Xu Zhou, Erin K O'Shea

**Keywords:** *S. cerevisiae*, Other

He BZ, Zhou X, O’Shea EK. 2017. Evolution of reduced co-activator dependence led to target expansion of a starvation response pathway. *eLife* **6**:e25157. doi: 10.7554/eLife.25157.

Published 9 May 2017

We were alerted by a user on PubPeer (link to post) to an error in Figure 1A, where we accidentally used the same phosphatase assay image for both *pho80∆ pho4∆* and *pho80∆ pho2∆* in *S. cerevisiae*. After carefully checking the original images and the figure raw file, we discovered that the error was due to the first author accidentally cropping the same row from the source images when assembling the figure. This mistake was not caught by the authors partly because the phosphatase assay phenotype of the two mutants were known to be highly similar (Kerwin and Wykoff 2009, PMID: 19332882, Figure 3). That being said, there is no excuse for the mistake of reusing the same image. The authors have now corrected the error by using the correct the image for each mutant. Importantly, the corrected results do not change any of the original conclusions. The corrected figure is shown below:

**Figure 1:**
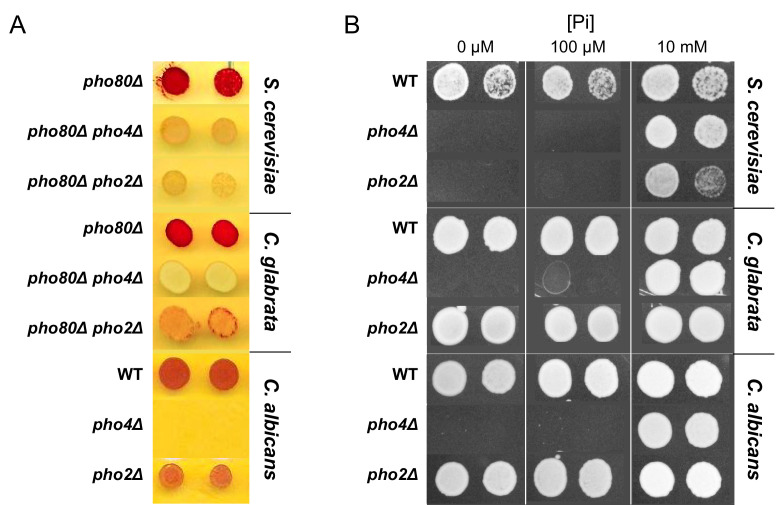


For comparison, the original figure is shown below, with the two rows of duplicated images highlighted in a red box.

**Figure 2:**
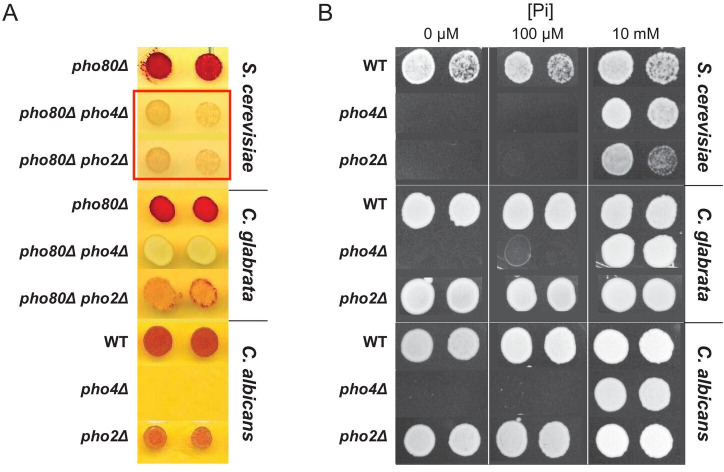


We also included the source image file for reference. In it, EY2851 corresponds to *pho80∆ pho2∆* and EY2852 corresponds to *pho80∆ pho4∆*. In the original figure 1A, we accidentally used the image for EY2851 twice for both mutants.

**Figure 3:**
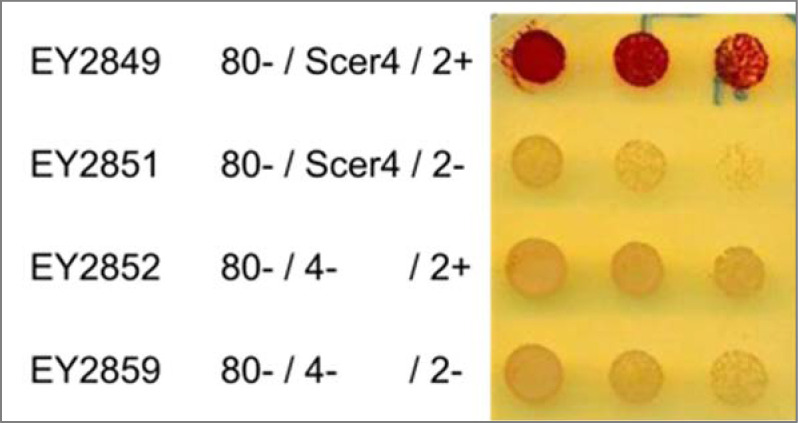


The article has been corrected accordingly.

